# IL-33 Alleviated Brain Damage via Anti-apoptosis, Endoplasmic Reticulum Stress, and Inflammation After Epilepsy

**DOI:** 10.3389/fnins.2020.00898

**Published:** 2020-08-31

**Authors:** Yuan Gao, Chengliang Luo, Yi Yao, Junjie Huang, Huifang Fu, Chongjian Xia, Guanghua Ye, Linsheng Yu, Junge Han, Yanyan Fan, Luyang Tao

**Affiliations:** ^1^Department of Forensic Science, Medical College of Soochow University, Suzhou, China; ^2^Department of Forensic Science, Wenzhou Medical University, Wenzhou, China; ^3^The Forensic Center, Wenzhou Medical University, Wenzhou, China; ^4^Center of Basic Medical Experiment, School of Basic Medical Science, Wenzhou Medical University, Wenzhou, China; ^5^Shanghai Key Laboratory of Forensic Medicine, Department of Shanghai Key Laboratory of Forensic Medicine, Shanghai Forensic Service Platform, Academy of Forensic Science, Shanghai, China; ^6^Department of Pathology, Traditional Chinese Medicine Hospital, Nanjing, China

**Keywords:** apoptosis, endoplasmic reticulum stress, interleukin-33, recurrent neonatal seizure, ST2

## Abstract

Interleukin (IL)-33 belongs to a novel chromatin-associated cytokine newly recognized by the IL-1 family, and its specific receptor is the orphan IL-1 receptor (ST2). Cumulative evidence suggests that IL-33 plays a crucial effect on the pathological changes and pathogenesis of central nervous system (CNS) diseases and injuries, such as recurrent neonatal seizures (RNS). However, the specific roles of IL-33 and its related molecular mechanisms in RNS remain confused. In the present study, we investigated the protein expression changes and co-localized cell types of IL-33 or ST2, as well as the effect of IL-33 on RNS-induced neurobehavioral defects, weight loss, and apoptosis. Moreover, an inhibitor of IL-33, anti-IL-33 was performed to further exploited underlying mechanisms. We found that administration of IL-33 up-regulated the expression levels of IL-33 and ST2, and increased the number of its co-localization with Olig-2-positive oligodendrocytes and NeuN-positive neurons at 72 h post-RNS. Noteworthily, RNS-induced neurobehavioral deficits, bodyweight loss, and spatial learning and memory impairment, as well as cell apoptosis, were reversed by IL-33 pretreatment. Additionally, the increase in IL-1β and TNF-α levels, up-regulation of ER stress, as well as a decrease in anti-apoptotic protein Bcl-2 and an increase in pro-apoptotic protein CC-3 induced by RNS are prevented by administration of IL-33. Moreover, IL-33 in combination with Anti-IL-33 significantly inverted the effects of IL-33 or Anti-IL-33 alone on apoptosis, ER stress, and inflammation. Collectively, these data suggest that IL-33 attenuates RNS-induced neurobehavioral disorders, bodyweight loss, and spatial learning and memory deficits, at least in part through mechanisms involved in inhibition of apoptosis, ER stress, and neuro-inflammation.

## Introduction

Epilepsy is one of the most common neurological diseases. Its symptoms are characterized by repetitive and persistent seizures, often accompanied by transient brain dysfunction caused by an abnormal synchronized discharge of important regions of the brain or the entire brain as well as emotional and cognitive dysfunction (Jones et al., 2010; Fisher, 2015). According to reports, the number of patients with epilepsy in China accounts for about one-fifth of the total number of patients with epilepsy in the world, with an annual increase of 400,000, and most of them are children (Berg et al., 2013). At present, there is still no better treatment plan for the treatment of epilepsy (van der Heide et al., 2012; Sousa, 2013). Antiepileptic drugs (ACDs) are still the main treatments, and other treatment methods such as surgery are supplemented (Enatsu and Mikuni, 2016). Due to the large toxic side effects of antiepileptic drugs and the immature surgical treatment, it is urgent to find effective measures to prevent and treat seizures.

The occurrence of epilepsy is a complicated pathological process, which mainly involves neuronal death, endoplasmic reticulum stress, and inflammatory response (Xiao et al., 2015). Apoptosis is one of the most important forms of neuronal death. The internal and external apoptosis pathways caused by the activation of caspase-8/10 and caspase-9, respectively, activate caspase-3, which leads to the occurrence of neuronal apoptosis (Sola-Riera et al., 2019). In particular, the endogenous apoptosis pathway plays a vital role in the occurrence and development of epilepsy (Henshall and Simon, 2005). The dynamin-related protein (Drp1) that belongs to a large GTPase of the dynamin superfamily is an intrinsic component of multiple mitochondria-dependent apoptosis pathways (Frank et al., 2001). It can promote cell apoptosis by using Bax to achieve mitochondrial outer membrane permeability and cytochrome c (Cyt-c) release from mitochondria (Cassidy-Stone et al., 2008). However, the inhibitor of Drp1 (mdivi-1) can exert neuroprotective effects against seizures-induced cell death of hippocampal neurons by inhibiting Cyt-c release, AIF translocation, and suppressing the mitochondrial apoptosis pathway (Zhou et al., 2020).

ERS is crucial for cellular homeostasis involved in protein synthesis and folding, protein trafficking, lipid, and sterol biosynthesis, and intracellular calcium regulation, and is the second major site for intrinsic apoptosis pathway (Yamamoto et al., 2006). Initiation ER could react responsively to various stresses (Paschen and Frandsen, 2001). A mild level of ERS activates a survival response (Liu M.Q. et al., 2016). However, severe or prolonged ERS subverts this response toward an apoptotic pathway (Gorman et al., 2012). Converging studies identified glucose-regulated protein 78 (GRP-78) and CATT/EBP homologous protein (CHOP) as ERS markers. GRP-78 belongs to a member of the heat shock protein 70 (HSP70) family, which is localized in the lumen of the endoplasmic reticulum (ER), and involved in the folding and assembly of proteins in the ER (Scott and McManus, 1999). As this protein interacts with many ER proteins, such as CHOP, it may play a key role in monitoring protein transport through the cell (Xie et al., 2016). Previous studies indicated seizure-induced brain insult is attenuated by the inhibition of GRP-78 and CHOP expression (Qiu et al., 2013). Moreover, an inhibitor of endoplasmic reticulum stress (salubrinal, SAL) relieves endoplasmic reticulum stress and exerts a neuroprotective effect by declining the expression levels of CHOP and caspase-3 proteins (Xie et al., 2016), indicating that inhibiting the endoplasmic reticulum stress response helps reduce the occurrence of apoptosis, thereby saving the death of nerve cells.

Besides, the inflammatory response plays an important role in the pathological changes of epilepsy. After epilepsy, increased expression levels of pro-inflammatory factors (IL-1β and TNF-α) can increase cell death, aggravate cerebral edema and worsen neurological dysfunction, while inhibiting the expression of these two inflammatory factors can alleviate brain damage (Figueiredo et al., 2019; Korotkov et al., 2020), indicating that inhibiting the inflammatory response plays a role in brain protection after seizures.

As a novel chromatin-related inflammatory factor, IL-33 induces target cells such as mast cells, NKT, and Th2 cells to produce various cytokines by binding to the specific receptor ST2 (Odegaard et al., 2016). There are two main types of ST2, including transmembrane ST2L and soluble ST2 (sST2). The function of ST2L is to transmit the signal of IL-33 and play a positive stimulatory role, while sST2 is deceptively combined with IL-33 to play a negative regulatory role (Iwahana et al., 1999; Allan et al., 2016). IL-33 is expressed and co-localized in many different types of cells, especially neurons and glia. IL-33 is released from apoptotic and necrotic cells as a vigilant signaling molecule (Palmer et al., 2009), and its synthesis, localization, and secretion are closely related to its dual functional properties. Indeed, IL-33 plays a double-edged sword in a variety of diseases, such as intracerebral hemorrhage (ICH) (Gao et al., 2017b), traumatic brain injury (TBI) (Gao et al., 2018) and central multiple sclerosis (MS) (Jafarzadeh et al., 2016). On the one hand, exogenous IL-33 can inhibit autophagic cell death and inflammatory responses, thereby protecting neurons from damage (Gao et al., 2017b). On the other hand, IL-33 promotes a large number of inflammatory cells infiltrating into brain tissue in MS, activating a severe inflammatory response, and exerting a neurotoxic effect (Jafarzadeh et al., 2016). Although IL-33’s roles have become increasingly clear, its precise role and underlying mechanisms in epilepsy have not been elucidated. Therefore, based on the above theoretical basis, we hypothesize that IL-33 as an important inflammatory regulator may alleviate RNS-induced neurobehavioral deficits, weight loss, and cell death. To investigate IL-33’s roles and underlying mechanisms, several novel agents including IL-33, Anti-IL-33 were used in our rat RNS model undergoing by which IL-33 regulates, apoptosis, ERS, and neuro-inflammation.

## Materials and Methods

### Animal Model, Reagents, and Experimental Groups

All animal experiments undertaken in this study were conducted according to an animal protocol approved by the Ethics Committee of Wenzhou Medical University. All procedures were in full compliance with the NIH Guide for the Care and Use of Laboratory Animals. All Sprague-Dawley (SD) rats were purchased from SLAC Company, Shanghai, China. Try to reduce the suffering and the number of animals used. The experimental procedure for establishing the RNS model had been described from our laboratory previously (Molofsky et al., 2015; Gao et al., 2017a). In Brief, on postnatal day (P7), SD rats (*n* = 48) were divided randomly into five groups: Sham + PBS group, RNS + PBS, RNS + IL-33 group, RNS + Anti-IL-33 group and RNS + Anti-IL-33 + IL-33 group. From P7, volatile flurothyl (Aldrich-Sigma, Chemical, United States) was used to induce recurrent seizures twice daily for 7 consecutive days in the rats the RNS group, with an interval time of 30 min once. Rats in the RNS group were injected intraperitoneally with recombinant mouse IL-33 (rmIL-33, 300 ng/rat, Biolegend, 580504), anti-IL-33 (300 ng/rat; R&D, AF3626) or PBS 30 min before the establishment of the RNS model, every other day for three times, respectively. However, the rats in the Sham + PBS group were placed in the container at the same time as the rats in the RNS + PBS group, and only the same amount of PBS was given. At 72 h (72 h) after the last flurothyl treatment, half of the rats were anesthetized with chloral hydrate and the brain was removed and stored at −80°C for further analyses.

### Double Immunofluorescent Staining

Standard immunofluorescent methods were applied for IL-33 IR cell types in the brains of Sham + PBS and RNS groups, which was described previously (Gao et al., 2017b). Brain tissue was fixed by cardiac perfusion, and the brain was first perfused with PBS, then perfused with 4% paraformaldehyde, and cut into 5 μm sections using a cryostat. Briefly, (1) the slices were fixed in 4% paraformaldehyde for 10 min; (2) Incubated with 3% H_2_O_2_ for 5–10 min at room temperature to eliminate endogenous peroxidase activity; (3) Washed with PBS three times for 5 min each time, and placed the slices in a boiled sodium citrate solution for microwave repair for 20 min; (4) 0.5% Triton punched for about 10min; (5) Washed PBS three times for 5 min each time, 5% BSA was blocked for 2 h; (6) Incubated sections with anti-IL-33 (1:100; R&D, AF3626), anti-ST2 (1:200; Abcam, ab25877), anti-NeuN (1:200; Abcam), anti-Olig-2 (1:500; Millipore) diluted in blocking buffer, then the sections were incubated for 2 h at 4°C with an appropriate fluorescence-conjugated secondary antibody (1:200, Jackson Immuno-Research). Sections were stained for DAPI (1:5000, Beyotime Institute of Biotechnology) to visualize the nucleus. Images were captured with a fluorescence microscope (Zeiss).

### Neurobehavioral Tests

Neurological behavioral parameters of brain damage (Open field test, forelimb suspension test, negative geotactic test, and cliff avoidance test)-induced by epilepsy were observed on P20 and P27, according to the procedure described previously (Ziegler et al., 2002; Zhang and Ney, 2008). (1) Open field test: Rats were placed in the center of the device (60 cm × 60 cm × 43 cm, 5 × 5 cells), and recorded the time that the rats spent in the center of the device as the delay time; The number of squares crossed that were crawled from the center of the device (all 4 limbs entered the same grid count) was recorded as the horizontal motion score, and the number of hind limbs upright (including the forelegs of the forelegs or the wall of the climbing box) was recorded as the vertical motion score. The sum of the players was the opening score. The number of squares crossed and the total time spent in the center of the device was quantified in 2 min bins over 10 consecutive min. Before each trial, the device was cleaned with 100% alcohol. (2) Forelimb suspension test: Allow Rats to grasp thin glass rods with their forepaws at time points P20 and P27, respectively, and record the time required for them to remain suspended only with the front paws. (3) Negative geotactic test: The rat’s head was placed down on a 45° angle ramp and the time it took to turn 180° and face the bevel was recorded. (4) Cliff avoidance test: Place the forelimbs of the rats on the edge of a 1.8 m high table and record the time they need to turn away from the edge.

### Morris Water Maze Test

The Morris water maze (MWM) experiment was mainly applied to the study of learning and memory mechanisms in RNS, according to the procedure described previously (Mychasiuk et al., 2015; Shin et al., 2015). In brief, for the place navigation test, during the five consecutive days (P28–P32), each rat was tested in the pool for 1 min to familiarize himself with the pool and the surrounding environment on P28. From P29 to P32, each rat was randomly placed in water in any quadrant of the non-target quadrant and allowed to find and climb the immersed platform within 60 s. Once the rat found the hidden platform submerged 2 cm under the water surface, it would be kept on the platform for an additional 10 s. Those rats that failed to find the submerged platform in the given time frame would be picked up platform in the given time frame would be picked up and placed on the platform for 10 s to identify spatial cues. Dry the rat’s hair with a hairdryer before being returned to the cage. The escape latency was automatically recorded by a video/computer system. For the spatial probe test, the submerged platform was removed from the pool on P34. Then allowed each rat to explore the pool within 60 s and the frequency of passing through the target quadrant was recorded by a video tracking system.

### Cytokine Enzyme-Linked Immune-Sorbent Assay (ELISA)

For quantification of the brain homogenates concentration of different cytokines isotypes, the corresponding Bethyl Mouse ELISA Quantification Kits (R&D) were used according to the manufacturer’s protocols. In brief, for the detection of IL-1β and TNF-α concentrations, samples, and standard dilutions were prepared. Added 100 μL of Standard, Control, or sample per assigned well. The plate frame was gently tapped for 1 min to ensure uniform mixing and incubation at 37°C for 1 h. The liquid in each well was aspirated, washed and patted dry, and the above procedure was repeated four times, and then 100 μL of mouse TNF-α or IL-1β conjugate was added to each well, and incubated at 37°C for 1 h. After washing and patted dry, 100 μL of the substrate solution was added to each well and incubated for 20 min in the dark, and then 100 μL of the stop solution was added to terminate the reaction. The absorbance was read at 450 nm using a microplate reader. The assay was performed in triplicate and the results are expressed as mean OD ± SEM.

### Real-Time PCR

Firstly, total RNA was extracted from rat brain tissue after RNS using Trizol reagent (Invitrogen). The RNA concentration was measured by NanoDrop 2000 (Thermo Fisher Scientific). Secondly, a total volume of 10 μl RNA samples was reverse transcribed at 42°C for 60 min and then incubated at 70°C for 5 min according to the product instructions (Thermo Fisher Scientific, Cat# #K1622). Finally, the reverse transcription product from the previous step was mixed in a reaction system with a total volume of 10 μl and subjected to PCR amplification. The reaction system included 2 μl cDNA, 5 μl mixed solution, 0.5 μl forward primer and 0.5 μl reverse primer, and 2 μl RNA-free water (Roche Life Science, Basel, Switzerland, Cat# 06924204001). PCR mixture was heated to 95°C for 10 min and cycled 40-45 times for each primer; cycles consisted of 95°C for 40 s, 53°C for 30 s, and 72°C for 40 s (LightCycler^®^ 96 System, Roche Life Science). Data shown are the relative abundance of the indicated mRNAs normalized to that of GAPDH. The sequences of the PCR primers for each gene are shown as follows:

IL-33, forward primer: 5′-AGGAAAGAACCCACGAAA-3′,reverse primer: 5′-GTCAACAGACGCAGCAAA-3′; GAPDH,forward primer: 5′-TATGTCGTGGAGTCTACTGGT-3′, reverseprimer: 5′-GAGTTGTCATATTTCTCGTGG -3′.

### Western Blot Analysis

Western blot (WB) analysis was performed using the standard methods previously described to detect protein levels of apoptosis, autophagy, and endoplasmic reticulum-associated proteins in each group of brains (Gao et al., 2017b). In brief, the supernatant containing the protein was extracted after being lysed the brain tissue, the protein concentration was adjusted, and the same amount of protein was separated by gel electrophoresis and transferred to a Hybond-polyvinylidene difluoride (PVDF) membrane. The PVDF membrane was then incubated with the primary antibodies to anti-NF-κB (1:500, CST), anti-ST2 (1:500, Abcam, ab25877), anti-IL-33 (1:500, R&D, AF3626), anti-Beclin-1 (1:1000, Bioworld), anti-LC3B (1:3000, Abcam), anti-P62 (1:1000, Abcam), anti-Bcl-2 (1:500, Abcam), anti-cleaved-caspase-3 (1:500, Bioworld) and anti-β-actin (1:10,000, Sigma). Anti-β-actin was used as a loading control. Then, the PVDF was incubated with the respective HRP-conjugated secondary antibody for 2 h at room temperature. Blots were detected with the ECL chemiluminescence system (Beyotime Institute of Biotechnology) and were captured on autoradiographic films (Kodak). Films were scanned and densitometric analysis of the bands was performed with Sigma Scan Pro 5.

### Statistics Analysis

All experiments were randomized independently and repeated in a blinded manner. One-way ANOVA with a Bonferroni test was used to assess the behavioral data and the frequency of the platform quadrant tested by the space probe. One-way ANOVA analysis followed by *post hoc* Tukey’s test and Dunnett *t*-test for multiple comparisons were used to analyze the data for assessing ELISA, RT-PCR, and western blot, respectively. Two-way analysis of variance (ANOVA; subject factor and time) for repeated measures were used to analyze data on weight gain and escape latency in the spatial probe test in the water maze. Results are expressed as mean ± standard error of the mean (SEM). For all two-two comparisons, *p* < 0.05 was considered statistically significant.

## Results

### The Expression Changes of IL-33 and ST2 Proteins After RNS

**FIGURE 1:**
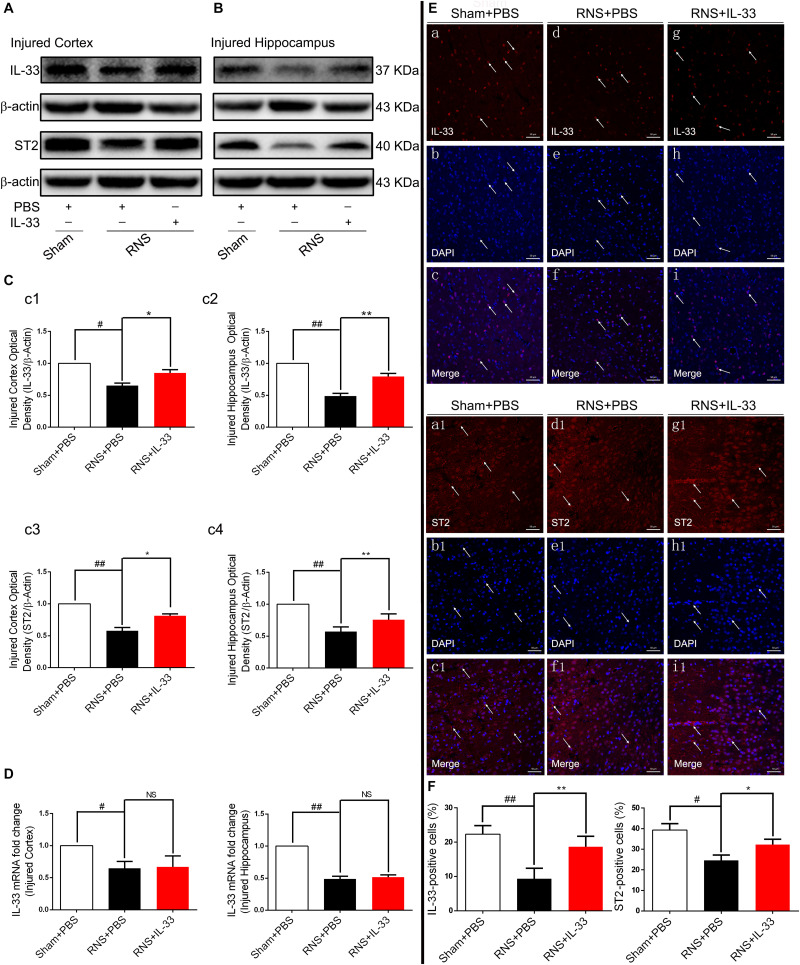
The changes of IL-33 and ST2 expression after RNS. **(A,B)** RNS induced a reduction of IL-33 and ST2 expression in both cortex and hippocampus tissues. **(C)** The optical densities of the two types of protein bands mentioned above were quantitatively analyzed. **(D)** Real-time PCR analysis of IL-33 mRNA in both cortex and hippocampus tissues. **(E)** Immunofluorescence staining was performed to demonstrate the changes of IL-33 and ST2 expression in cortex tissue. Bar 50 μm. **(F)** Semi-quantitative analysis of IL-33 or ST2 positive cells relative to the total number of cells. The data were expressed as means ± SEM (*n* = 6). ^##^*P* < 0.01 vs. Sham + PBS group, ^#^*P* < 0.05 vs. Sham + PBS group. **P < 0.01 vs. RNS + PBS group, *P < 0.05 vs. RNS + PBS group. No significance (NS) was observed between RNS + IL-33 group and RNS + PBS group. Experiments are representative of three independent experiments.

To investigate the role of IL-33 in RNS, we firstly examined the dynamic changes of IL-33 and its receptor-specific ST2 protein in the cortex and hippocampus after RNS (Figures 1A–C). The results showed that the mRNA and protein of IL-33 and ST2 were highly expressed in the Sham + PBS group. RNS induced a significant down-regulation of their mRNA and protein expression levels, compared with that of Sham + PBS group (*P* < 0.05; *P* < 0.01). IL-33 pretreatment caused a significant increase in the protein levels of IL-33 and ST2 (*P* < 0.05; *P* < 0.01), but did not significantly change IL-33 mRNA level in cortex and hippocampus (*P* > 0.05, Figure 1D), indicating that the upregulation of IL-33 protein level is due to exogenous supplementation of IL-33. To further clarify the experimental results of the above-mentioned WB, immunofluorescent staining was used to evaluate the expression changes of the above two proteins. Consistent with the results of WB, the results of immunofluorescence suggested that IL-33 pretreatment led to an increase in IL-33 and ST2 expressions, and they are mainly localized in the nucleus and membrane of cerebral cells, respectively (*P* < 0.05; *P* < 0.01, Figures 1E,F), suggesting that exogenous IL-33 has been arrived at the site of the injured brain parenchyma and may play a relevant role in RNS.

### The Expression and Co-localization of IL-33 in RNS

**FIGURE 2:**
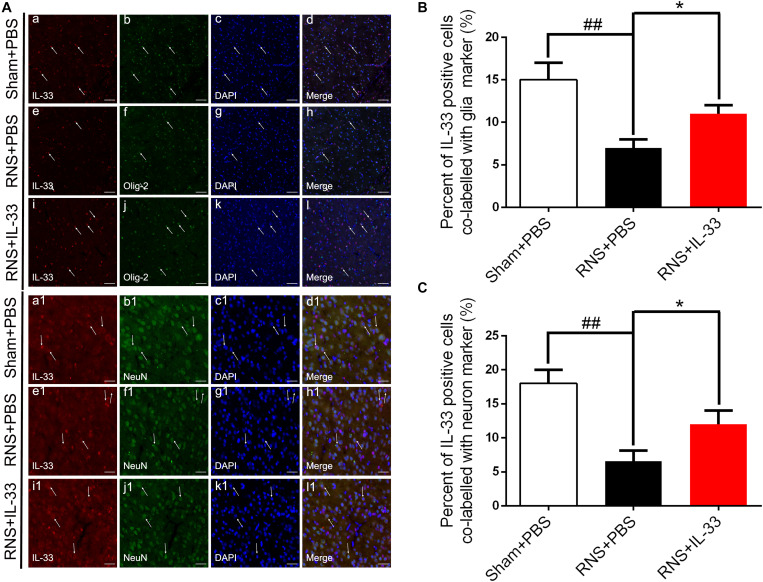
The expression and co-localization of IL-33 in cerebral cells and tissues after RNS. **(A)** Representative images of double labeling were indicated with white arrows after RNS. **(a-l)** The co-localization of IL-33-like immunoreactivity and Olig-2-positive. Bar 50 μm. **(a1–l1)** Co-localization of IL-33-like immunoreactivity and NeuN-positive neurons. Bar 50 μm. **(B,C)** Semi-quantitative analysis of glia and neuron type-cells contributions to the IL-33-positive cell population. The data were expressed as means ± SEM (*n* = 6). ^##^*P* < 0.01 vs. Sham + PBS group. *P < 0.05 vs. RNS + PBS group. Experiments are representative of three independent experiments.

To investigate the cell types expressed by IL-33 after RNS, we examined the co-labeling of IL-33 with specific markers for oligodendrocyte (Olig-2) (Figures 2Aa–l,B) and neuron (NeuN) (Figures 2Aa1–l1,C), respectively. We found that IL-33 was highly expressed in Olig-2-positive oligodendrocyte and NeuN-positive neurons of the normal group, and mainly localized in the nucleus of oligodendrocyte and cytoplasm of neurons. Compared with that of Sham + PBS group, the RNS group contributed to a significant reduction in the positive percentage of the two types of double-labeled cells (*P* < 0.01). However, the administration of IL-33 reversed RNS-induced the decrease in the positive percentage of the two types of double-labeled cells mentioned above (*P* < 0.05).

### IL-33 Improves RNS-Induced Neurobehavioral Defects and Promotes Weight Recovery

**FIGURE 3:**
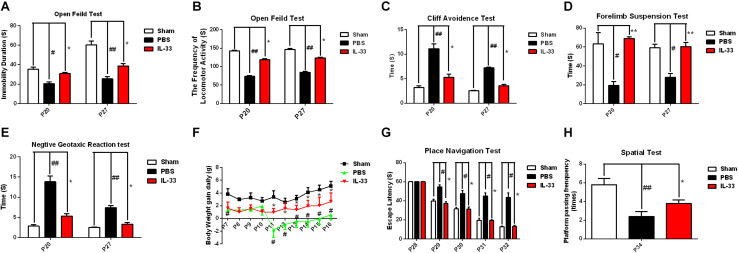
IL-33 alleviated neurologic behavioral deficits, bodyweight loss, and performance in the Morris water maze (MWM) test after RNS. **(A–E)** The time of each group in the open field test, forelimb suspension test, negative geotactic test, and cliff avoidance test was recorded at P20 and P27, respectively. **(F)** IL-33 reversed RNS-induced the reduction in BWG during P7-P16. **(G)** In the MWM test, mean escape latency for each group was plotted during P28–P32. **(H)** The frequencies of crossing the platform were recorded at P34. The data were expressed as means ± SEM (*n* = 6). ^##^*P* < 0.01 vs. Sham + PBS group, ^#^*P* < 0.05 vs. Sham + PBS group. **P < 0.01 vs. RNS + PBS group, *P < 0.05 vs. RNS + PBS group. Experiments are representative of three independent experiments.

To assess the effect of IL-33 on neurologic development and bodyweight gain (BWG), different neurological tests were present in Figures 3A–E. The results showed that the RNS + PBS group caused an evident delay or reduction in forelimb suspension test, negative geotactic reaction test, cliff avoidance test, and open field test, compared with the Sham + PBS group (*P* < 0.05; *P* < 0.01). In contrast, IL-33 pretreatment reversed these behavioral deficiencies, compared with the RNS + PBS group (*P* < 0.05; *P* < 0.01). Besides, the temporal changes of BWG were detected from P7 to P16 (Figure 3F). The results indicated that the RNS group led to a significant reduction in BWG at P7. From P11 to P15, the BWG presented a negative increase and reached the valley at P11. Subsequently, the reduction in BWG gradually returned to baseline levels but remained the decreased levels for up to P16 (*P* < 0.05). However, IL-33 administration reversed the bodyweight loss caused by RNS, suggesting that IL-33 may contribute to the recovery of post-epileptic bodyweight loss (*P* < 0.05).

### IL-33 Ameliorated Performance in Morris Water Maze (MWM) Test After RNS

To investigate whether IL-33 has effect on learning and memory impairment after epilepsy, the experiments of water maze and navigational navigation were applied to this study (Figures 3G,H). The results showed that significantly longer in the escape latencies of MWM were found in the RNS group from P28 to P32 than that in the Sham + PBS group (*P* < 0.05), however, the IL-33 pretreatment group significantly reduced the latency compared with the RNS + PBS group (*P* < 0.05). As for the spatial probe test, the RNS + PBS group contributed to a significant lower in the frequency of passing through the platform quadrant than that in the Sham + PBS group (*P* < 0.01), whereas, IL-33 pretreatment markedly increased the probe tests compared with the RNS group (*P* < 0.05).

### IL-33 Inhibited RNS-Induced Inflammatory Responses and NF-κB Activity

**FIGURE 4:**
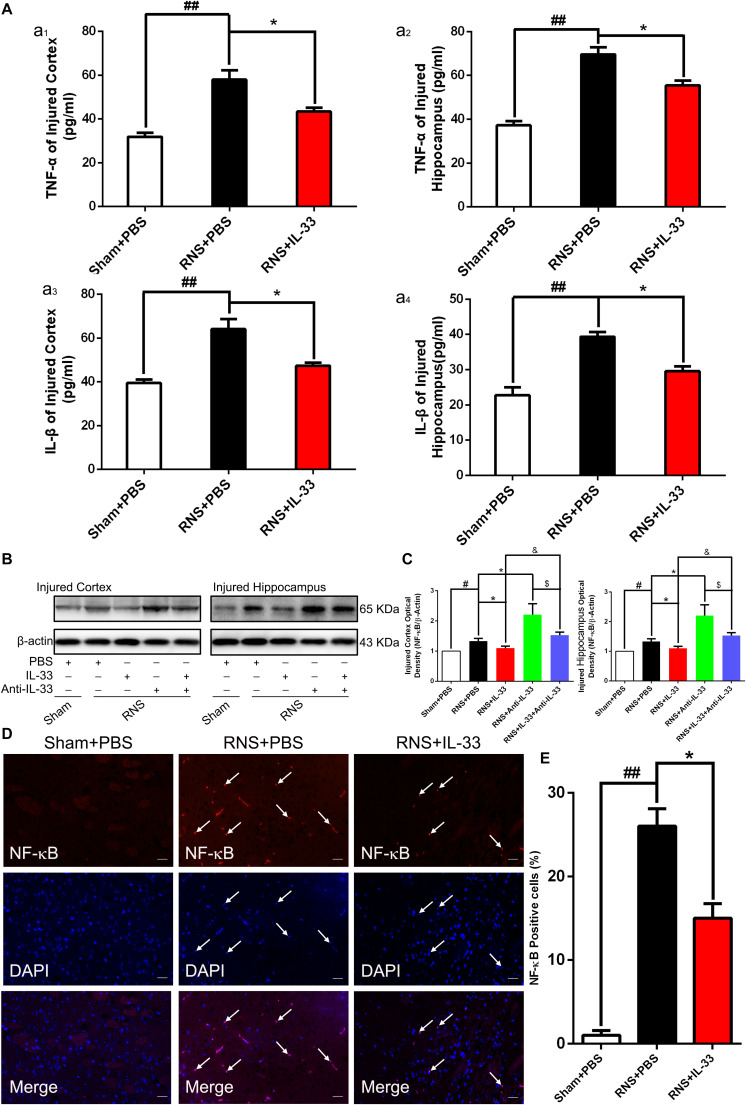
IL-33 inhibited NF-κB-mediated inflammatory responses after RNS. **(A)** ELISA was employed to assess the expression levels of IL-1β or TNF-α at 72 h post-RNS. RNS-induced increase in TNF-α and IL-1β expression levels were reversed by IL-33 pretreatment in the brain cortex (a1 and a3) and hippocampus tissues (a2 and a4) after a seizure. **(B)** An increase in NF-κB activity caused by RNS was detected. Administration of IL-33 alone down-regulated the expression level of NF-κB, while Anti-IL-33 alone treatment up-regulated the expression level of NF-κB. However, the combined treatment of IL-33 and Anti-IL-33 reversed the effect of IL-33 alone or Anti-IL-33 on the expression level of NF-κB, indicating that IL-33 inhibited the activity of NF-κB at 72 h post-RNS, suggesting that IL-33 possesses a capacity to inhibit NF-κB activity. **(C)** The optical densities of the protein bands were quantitatively analyzed, and normalized with loading control β-actin. **(D)** IL-33 treatment reduces nuclear NF-κB expression. **(E)** Semi-quantitative analysis of NF-κB-positive cells relative to the total number of cells. The data were expressed as means ± SEM (*n* = 6). ^##^*P* < 0.01 vs. Sham + PBS group, ^#^*P* < 0.05 vs. Sham + PBS group. *P < 0.05 vs. RNS + PBS group. ^&^*P* < 0.05 vs. RNS + IL-33 group.^ $^*P* < 0.05 vs. RNS + Anti-IL-33 group Experiments are representative of three independent experiments.

To demonstrate the effect and underlying mechanisms of IL-33 in inflammatory responses after RNS. ELISA and WB were carried out to assess the level of inflammatory cytokines and NF-κB expression, respectively (Figure 4). These results indicated that an evident up-regulation in TNF-α (Figures 4Aa1,a2) and IL-1β (Figures 4a3,a4) expression was found in both cortex and hippocampus of RNS group at 72 h after RNS (*P* < 0.01). However, the administration of IL-33 reversed the up-regulation of IL-1β and TNF-α levels (*P* < 0.05).

After epilepsy, the activity of NF-κB is positively correlated with the expression of inflammatory factors, and inhibition of NF-κB expression plays a role in inhibiting inflammatory response (Yu et al., 2013). Here, we observed that a remarkable increase of NF-κB activity induced by RNS in both cortex and hippocampus at 72 h after RNS, compared with that of Sham + PBS group (*P* < 0.05). However, pretreatment with IL-33 significantly inhibited NF-κB activity, compared with the RNS + PBS group (*P* < 0.05; *P* < 0.01). IL-33 in combination with Anti-IL-33 remarkably increased the expression level of NF-κB compared with the IL-33 group. To further verify the effect of endogenous IL-33 on NF-κB activity after RNS, Anti-IL-33 alone and combined IL-33 treatments were used in this experiment. We found that Anti-IL-33 treatment alone significantly increased the expression level of NF-κB, compared to the RNS + PBS group. By contrast, IL-33 in combination with Anti-IL-33 markedly reduced the expression level of NF-κB compared with Anti-IL-33 group (*P* < 0.05; *P* < 0.01, Figures 4B,C), which indicated IL-33 plays a role in inhibiting NF-κB activity after RNS.

Furthermore, to explore whether Il-33 inhibits the expression of NF-κB in the nucleus, dual-label immunofluorescence staining of NF-κB and DAPI was applied in the study (Figures 4D,E). The recent results showed that RNS + PBS induced an increase in NF-κB nuclear expression, compared with that of Sham + PBS group. Administration of IL-33 remarkably reduced the nuclear expression of NF-κB, compared with the RNS + PBS group, suggesting that IL-33 down-regulated the expression level of NF-κB in the nucleus.

### IL-33 Treatment Suppressed RNS-Induced Cell Apoptosis After RNS

**FIGURE 5:**
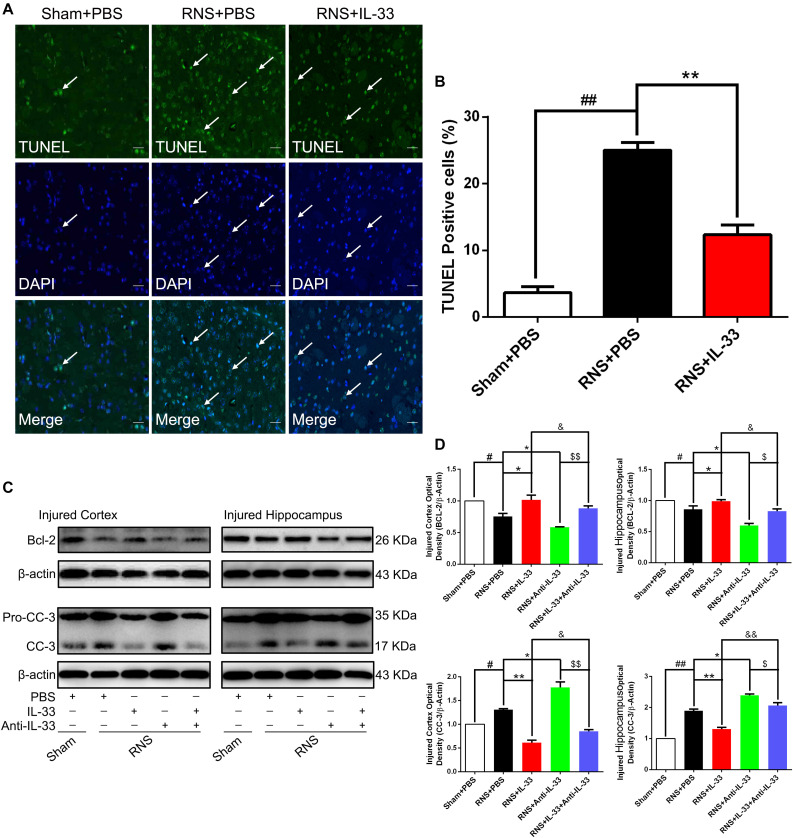
IL-33 inhibited cell apoptosis through reversing RNS-induced Bcl-2 decrease and CC-3 increase. **(A)** Representative immunofluorescence photomicrograph showed a significant increase in the number of TUNEL-positive cells. However, the administration of IL-33 contributed to a significant decrease in TUNEL-positive cells at 72 s post-RNS. **(B)** Semi-quantitative analysis of TUNEL-positive cells relative to the total number of cells. **(C)** Administration of IL-33 alone reversed RNS-induced down-regulation of Bcl-2 expression and up-regulation of CC-3 expression, while Anti-IL-33 alone treatment further down-regulated the expression level of Bcl-2 and up-regulated the expression level of CC-3. However, the combined treatment of IL-33 and Anti-IL-33 reversed the effect of IL-33 alone or Anti-IL-33 on the expression level of Bcl-2 and CC-3, suggesting that IL-33 inhibits RNS-induced apoptosis at 72 h post-RNS. IL-33 pretreatment and Anti-IL-33 treatment reversed IL-33’s effect on the two types of apoptosis-related proteins in both cortex and hippocampus tissues after RNS. **(D)** Optical densities of the protein bands were quantitatively analyzed, and normalized with loading control β-actin. The data were expressed as means ± SEM (*n* = 6). **^##^***P* < 0.01 vs. Sham + PBS group, **^#^***P* < 0.05 vs. Sham + PBS group. **P < 0.01 vs. RNS + PBS group, *P < 0.05 vs. RNS + PBS group. ^&&^*P* < 0.01 vs. RNS + IL-33 group, ^&^*P* < 0.05 vs. RNS + IL-33 group.^ $$^*P* < 0.01 vs. RNS + IL-33 group, ^$^*P* < 0.05 vs. RNS + Anti-IL-33 group. Experiments are representative of three independent experiments.

To determine whether IL-33 can reduce RNS-induced apoptosis, TUNEL staining was performed to assess apoptotic cell death (Figures 5A,B). Our results indicated that RNS robustly increased the number of TUNEL-positive cells at 72 h post-RNS, compared with the Sham + PBS group (*P* < 0.01). However, IL-33 pretreatment remarkably decreased that of the above TUNEL-positive cells (*P* < 0.01). The data suggest that IL-33 can inhibit RNS-induced apoptosis after RNS.

### IL-33 Treatment Up-Regulated the Level of Bcl-2 Expression After RNS

Bcl-2 family proteins are gatekeepers of endogenous apoptosis, especially Bcl-2. And its increased expression can inhibit cell apoptosis in a variety of disease models. To investigate the underlying mechanism of IL-33 reducing RNS-induced apoptosis, western blotting was applied to assess the level of Bcl-2 expression (Figures 5C,D). We found that Bcl-2 was highly expressed in the Sham + PBS group, but RNS significantly induced the decrease of Bcl-2 expression (*P* < 0.05; *P* < 0.01). However, IL-33 pretreatment inhibited down-regulation of Bcl-2 expression in both cortex and hippocampus at 72 h after RNS (*P* < 0.05; *P* < 0.01). IL-33 in combination with Anti-IL-33 remarkably down-regulated the expression level of Bcl-2 compared with the IL-33 group. To further demonstrate whether endogenous IL-33 plays an anti-apoptotic effect on RNS, Anti-IL-33 alone and combined IL-33 treatments were used in this experiment. We found that Anti-IL-33 treatment alone significantly down-regulated the expression level of Bcl-2 compared with the RNS + PBS group. On the contrary, IL-33 in combination with Anti-IL-33 markedly up-regulated the expression level of Bcl-2 compared with the Anti-IL-33 group (*P* < 0.05; *P* < 0.01), which indicated IL-33 played a role in inhibiting apoptosis after RNS.

### IL-33 Treatment Suppressed Cleaved-Caspase (CC)-3 Expression After RNS

To investigate the role and underlying mechanism of IL-33 in the apoptotic pathway after RNS, Western blotting was utilized to evaluate the level of CC-3 expression (Figures 5C,D). We found that RNS significantly induced the increase of CC-3 expression, compared with the Sham + PBS group (*P* < 0.05). However, IL-33 pretreatment inhibited the up-regulation of CC-3 expression in both cortex and hippocampus at 72 h after RNS (*P* < 0.05). IL-33 in combination with Anti-IL-33 remarkably down-regulated the expression level of CC-3 compared with IL-33 group. Besides, to further determine whether endogenous IL-33 plays an anti-apoptotic effect on RNS, Anti-IL-33 alone and combined IL-33 treatments were used in this experiment. We found that Anti-IL-33 treatment alone significantly increased the expression level of CC-3 compared to the RNS + PBS group. By contrast, IL-33 in combination with Anti-IL-33 markedly reduced the expression level of CC-3 compared with the Anti-IL-33 group (*P* < 0.05; *P* < 0.01), which indicated IL-33 could inhibit RNS-induced apoptosis.

### IL-33 Treatment Suppressed Drp 1 Expression After RNS

**FIGURE 6:**
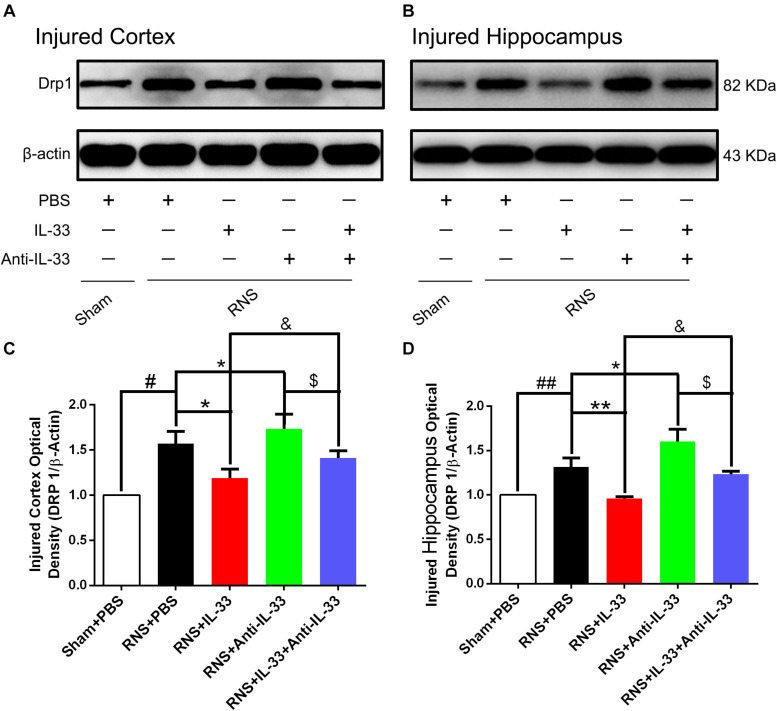
IL-33 reversed RNS-induced the increase of Drp 1 after RNS. **(A,B)** Administration of IL-33 alone reversed RNS-induced up-regulation of Drp 1 expression level, while Anti-IL-33 alone treatment further down-regulated the expression level of Drp 1. However, the combined treatment of IL-33 and Anti-IL-33 reversed the effect of IL-33 alone or Anti-IL-33 on the expression level of Drp 1 in both cortex and hippocampus at 72 h post-RNS. **(C,D)** Optical densities of the protein bands were quantitatively analyzed, and normalized with loading control β-actin. The data were expressed as means ± SEM (*n* = 4–6). ^##^*P* < 0.01 vs. Sham + PBS group, ^#^*P* < 0.05 vs. Sham + PBS group. **P < 0.01 vs. PBS group, *P < 0.05 vs. PBS group. ^&^*P* < 0.05 vs. RNS + IL-33 group.^ $^*P* < 0.05 vs. RNS + Anti-IL-33 group. Experiments are representative of three independent experiments.

Drp1 activation involves mitochondrial translocation, leading to mitochondrial fission or fragmentation. As shown in Figure 6, we found that RNS significantly induced the increase of Drp1 expression, compared with the Sham + PBS group (*P* < 0.01). However, IL-33 pretreatment inhibited the up-regulation of Drp 1 expression in both cortex and hippocampus at 72 h after RNS (*P* < 0.01). IL-33 in combination with Anti-IL-33 remarkably down-regulated the expression level of Drp 1 compared with the IL-33 group. Besides, to further determine whether IL-33 plays an anti-apoptotic effect on RNS, Anti-IL-33 alone, and combined IL-33 treatments were used in this experiment. We found that Anti-IL-33 treatment alone significantly increased the expression level of Drp1 compared to the RNS + PBS group, while Anti-IL-33 combined with IL-33 treatment markedly reduced the expression level of Drp1 compared with the Anti-IL-33 group (*P* < 0.05; *P* < 0.01), which indicated IL-33 inhibited apoptosis by reducing the expression level of Drp1 after RNS.

### IL-33 Treatment Reversed RNS-Induced Endoplasmic Reticulum Stress (ERS)

**FIGURE 7:**
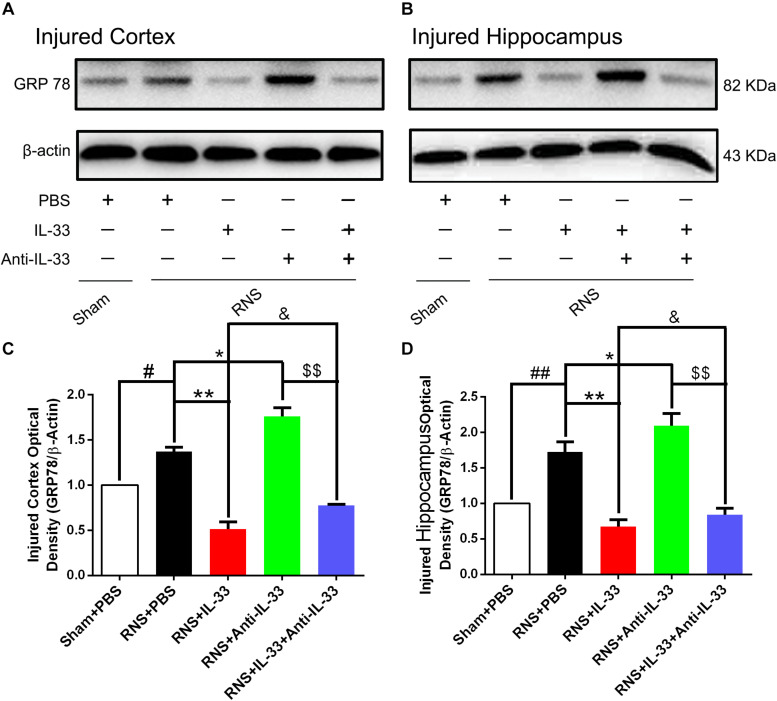
IL-33 negatively regulated RNS-induced ERS responses. **(A,B)** Administration of IL-33 alone reversed RNS-induced up-regulation of the expression level of ERS related-protein GRP-78, while Anti-IL-33 alone treatment further up-regulated the expression level of GRP-78. However, the combined treatment of IL-33 and Anti-IL-33 reversed the effect of IL-33 alone or Anti-IL-33 on the expression level of GRP-78 in both cortex and hippocampus at 72 h post-RNS. **(C,D)** Optical densities of the protein bands were quantitatively analyzed, and normalized with loading control β-actin. The data were expressed as means ± SEM (*n* = 4–6). ^##^*P* < 0.01 vs. Sham + PBS group, ^#^*P* < 0.05 vs. Sham + PBS group. **P < 0.01 vs. PBS group, *P < 0.05 vs. PBS group. ^&^*P* < 0.05 vs. RNS + IL-33 group.^ $$^*P* < 0.01 vs. RNS + Anti-IL-33 group. Experiments are representative of three independent experiments.

To investigate the role of IL-33 in ERS after RNS, western blotting was performed to detect the expression levels of endoplasmic reticulum stress-related protein (GRP-78). As shown in Figure 7, RNS significantly led to the increase of GRP-78 expression, compared with the Sham + PBS group (*P* < 0.05). By contrast, IL-33 pretreatment inhibited the up-regulation of GRP-78 expression, compared with the RNS + PBS group (*P* < 0.05; *P* < 0.01). IL-33 combined with Anti-IL-33 treatment remarkably down-regulated the expression level of GRP-78 compared with the IL-33 group. Besides, to further verify whether endogenous IL-33 has an inhibitory effect on ERS after RNS, Anti-IL-33 alone and combined IL-33 treatments were used in this experiment. We found that Anti-IL-33 treatment alone significantly increased the expression level of GRP-78 compared to the RNS + PBS group, while Anti-IL-33 combined with IL-33 treatment remarkably increased the expression level of GRP-78 compared with IL-33 group; markedly reduced the expression level of GRP-78 compared with Anti-IL-33 group (*P* < 0.05; *P* < 0.01), which indicated IL-33 played a role in inhibiting ERS after RNS.

## Discussion

In the present study, we demonstrated that RNS significantly resulted in a reduction of IL-33 and ST2 expression in the cortex and hippocampus tissues, and IL-33 was mainly co-localized in the nucleus of oligodendrocyte and cytoplasm of neurons. IL-33 administration improved neurobehavioral deficits, bodyweight loss, and cell apoptosis caused by RNS. Intriguingly, the neuroprotective mechanisms of IL-33 in RNS was through down-regulating the expression levels of IL-1β, TNF-α, NF-κB, CC-3, Drp-1, and GRP-78, as well as up-regulating that of Bcl-2. Moreover, IL-33 in combination with Anti-IL-33 reversed IL-33 ‘s neuroprotective effects on apoptosis, ER stress, and inflammation. Taken together, IL-33 provided a neuroprotective effect, at least in part, by inhibiting apoptosis, ER stress, and neuroinflammation at 72 h post-RNS.

Our current results showed that RNS induced a significant reduction of IL-33 and ST2 was observed in both the cortex and hippocampus after RNS, but exogenous IL-33 inverted the down-regulation of IL-33 and ST2 expression. Further research at the genetic level revealed that the expression level of IL-33 mRNA was significantly down-regulated after RNA, but exogenous IL-33 did not affect its mRNA expression level, indicating that exogenous IL-33 might reach the injured brain to make up for the insufficiency of IL-33 expression. Numerous studies have suggested that IL-33 is not only expressed in Olig-2 positive oligodendrocytes, but it is also one of the main cell types derived from IL-33 (Zarpelon et al., 2016). Besides, IL-33 and ST2 are both expressed in neurons (Jiang et al., 2012; Liu B. et al., 2016), and the transduction of IL-33 signaling pathway depends on ST2 (Yang et al., 2017). Furthermore, it is well known that the characteristic pathological changes of seizures are abnormal neuronal discharge, accompanied by the loss of large numbers of neurons and the death of glial cells. Then, the possible reasons for the decrease in IL-33 induced by RNS may be given as follows: (1) The death of neurons and glial cells occurs; (2) ST2 forms a complex with IL-33 or sST2 decoy binds IL-33; (3) IL-33 released by apoptosis and necrosis of nerve cells reaches the peripheral blood through the blood-brain barrier.

To date, the expression and localization of IL-33 and ST2 in the central nervous system (CNS) remain controversial. Numerous literature identified that IL-33 was produced by endothelial cells and astrocytes but not by microglia or neurons, and its receptor, ST2, was mainly expressed in microglia and astrocytes cultured *in vitro* (Huang et al., 2015). Other reports revealed that IL-33 and ST2 were mainly localized in astrocytes, microglia, and neurons, as well as oligodendrocytes after brain injury (Jiang et al., 2012; Allan et al., 2016; Gao et al., 2017a). Consistent with the latter, our current results showed that IL-33 was expressed at high levels in the Sham group and almost exclusively co-localized in the nucleus of oligodendrocytes, and the cytoplasm of neurons. RNS led to a remarkable decrease in the two types of double-labeled cells mentioned above. However, IL-33 administration reversed the reduction of them. Previous reports indicated IL-33 was mainly derived from Olig-2-positive oligodendrocytes in neuropathic pain (Zarpelon et al., 2016). Whereas, in EAE, IL-33 was primarily located in NeuN-positive neurons (Jiang et al., 2012), suggesting the source of IL-33 may be related to the type of disease. Additionally, when cells underwent apoptosis or necrosis, IL-33 was released into the cytoplasm and interstitial through a paracrine or autocrine pathway (Sangiuliano et al., 2014). Subsequently, IL-33 bound to its specific cell membrane receptor ST2, causing the conduction of downstream signals (Kakkar and Lee, 2008). Therefore, the contradiction of the cell types expressed by IL-33 may be due to different disease models, experimental settings, *in vitro* and *in vivo* environments, or due to the tissue repair process after RNS. This interesting observation deserves further investigation.

Different expression and localization of IL-33 and ST2 may affect their functions and mechanisms of action. Multiple types of cell death, such as apoptosis, autophagic cell death, and necroptosis can be induced by seizures (Lopez-Meraz et al., 2010; Benz et al., 2014). Apoptosis is the most important form of death of nerve cells after epilepsy, which has several typical features including cell shrinkage, chromatin condensation, DNA degradation, apoptotic body formation, and various mechanisms involved in epilepsy. Post-programmed cell death, including endogenous and exogenous pathways caused by caspase-8/10 and caspase-9 activation, respectively (Yakovlev and Faden, 2001), which can activate caspase-3, ultimately lead to the occurrence of neuronal apoptosis. Indeed, status epilepticus could contribute to neuronal apoptosis accompanied by producing a subset of TUNEL-positive neurons in up to 20 of 23 brain regions at 72 h post-RNS (Fujikawa et al., 2000). Moreover, effective inhibition of neuronal apoptosis provided a neuroprotective effect by the up-regulation of Bcl-2, Bcl2l1, Bdnf, Sox-2, and NeuroD1 genes in an animal model of temporal lobe epilepsy (Corvino et al., 2012). Noteworthily, less is known about the role and underlying mechanisms of IL-33 in the epilepsy model. Our previous studies suggested that IL-33 could exert a neuroprotective role by regulating the Bcl-2/CC-3 signaling pathway after RNS (Gao et al., 2017a). Consistent with previous studies, our current results revealed that IL-33 administration reduced the number of TUNEL-positive cells at 72 h post-RNS, suggesting that IL-33 processes the anti-apoptotic effect. However, IL-33’s related mechanisms in apoptosis after RNS is still confused. Numerous studies suggested that the mechanism of neuronal apoptosis induced by epilepsy is related to intrinsic and extrinsic apoptotic pathways, endoplasmic reticulum stress response, and inflammatory response (Yamamoto et al., 2006; Engel et al., 2010; Vince and Silke, 2016). The dynamin-related protein Drp1 which belongs to a large GTPase of the dynamin superfamily is a key protein to induce mitochondrial fission, and ultimately induce apoptosis (Cassidy-Stone et al., 2008). It is also required to achieve mitochondrial outer membrane permeability (MOMP) with the pro-apoptotic protein Bax, and Cyt-c release from mitochondria (Cassidy-Stone et al., 2008). Moreover, an inhibitor of Drp1, mdivi-1 could significantly rescue neurons from death induced by seizures in a dose-dependent manner via inhibiting Cyt-c release, AIF translocation, and suppressing the mitochondrial apoptosis pathway (Brooks et al., 2011). Intriguingly, our results revealed that IL-33 pretreatment significantly down-regulated the levels of Drp1, CC-3 expression, and maintained Bcl-2 high level at 72 h post-RNS. We suspected that inhibition of Drp1 expression might indirectly or directly attenuate the pro-apoptotic effect of Bax, in turn reducing the release of Cyt-c, thereby causing down-regulation of CC-3, ultimately inhibiting the occurrence of apoptosis, or through increasing the expression of BCL-2. The latter can form a complex with Bax through the BH3 binding domain, thereby down-regulating free Bax and ultimately inhibiting apoptosis.

Additionally, the endoplasmic reticulum has emerged as an important instigator of the intrinsic apoptotic pathway, involved in several neurodegenerative and neurological disorders, including temporal lobe epilepsy (Paschen and Mengesdorf, 2005; Yamamoto et al., 2006). It is very sensitive to changes in the environment, and any changes in the internal equilibrium state may cause activation of the endoplasmic reticulum stress response. When ER stress occurs, the dissociation of glucose regulatory protein 78 (GRP-78) activates and triggers an unfolded protein response (UPR). UPR is an adaptive response to the restored normal ER function. And ERS-mediated cell damage is mainly related to the endoplasmic reticulum UPR. In ERS, UPR protects cells by down-regulating translation and up-regulating endoplasmic reticulum chaperone molecules, thereby reducing ERS. However, excessive and prolonged ER stress triggers the activities of CHOP and caspase-12, and then activates caspase-9 and caspase-3 to induce apoptosis (Ljubkovic et al., 2019). By contrast, endoplasmic reticulum stress inhibitors (salubrinal, SAL) can offer neuroprotective effects through suppressing endoplasmic reticulum stress responses accompanied by decreasing CHOP and caspase-3 protein expression after brain injury (Logsdon et al., 2016). Besides, SAL treatment can promote neuronal regeneration and reduce mature neurons by inhibiting phosphorylation of eIF2α and ATF4, ultimately improving memory impairment after traumatic brain injury (Rubovitch et al., 2015), which shows that effective inhibition of endoplasmic reticulum stress does have a neuroprotective role. Our results showed that IL-33 pretreatment down-regulated the expression level of endoplasmic reticulum stress-related protein GRP-78, while Anti-IL-33 combined with IL-33 treatment reversed the role of IL-33 alone in inhibiting endoplasmic reticulum stress after RNS. The above shreds of evidence suggested that IL-33 might exert a neuroprotective effect of inhibiting apoptosis by inhibiting mitochondrial division and endoplasmic reticulum stress after epilepsy.

Besides the mechanisms mentioned above, inflammatory responses can also lead to apoptosis. To our acknowledge, the inflammatory response is a double-edged sword. Appropriate inflammation can play a defensive role against brain tissue damage, while the excessive inflammatory response has a deteriorating effect on brain tissue damage (Russo and McGavern, 2016). Previous studies showed seizure activity led to the production of inflammatory molecules including IL-1β and TNF-α (He et al., 2020). In turn, IL-1β or TNF-α could contribute to the severity and relapse of seizures (Yue et al., 2020). Moreover, inhibition of IL-1β or TNF-α expression may lead to prevention or delay of seizures and play a neuroprotective effect on a rat model of temporal lobe epilepsy (Noe et al., 2013; Sitges et al., 2014). In the study, we found that IL-33 pretreatment inhibited the inflammatory response by down-regulating the expression levels of IL-1β and TNF-α after RNS, indicating that IL-33 plays an anti-inflammatory role after RNS. However, the mechanism of how IL-33 regulates the expression of these two pro-inflammatory factors is unclear. Numerous studies revealed that various mechanisms are involved in triggering the inflammatory response after RNS, especially the NF-κB signaling pathway. Previous studies have demonstrated that NF-κB signaling is closely related to the development and progression of inflammatory responses primarily by mediating the synthesis of proinflammatory cytokines and chemokines. The activation of NF-κB can trigger various pro-inflammatory genes, such as IL-1β and TNF-α. In turn, pro-inflammatory factors can also activate NF-κB through autocrine and paracrine pathways, thereby forming a positive circulation pool between them. On the contrary, effective inhibition of NF-κB activation can down-regulate the level of cytokines, thereby blocking excitatory neuron death, reducing BBB permeability, and improving neurological deficits (Grilli et al., 1996), suggesting that inhibition of NF-κB activation and the inflammatory response has certain brain-protective effects after SE. The current results showed that administration of IL-33 reduced the activity of NF-κB, and down-regulated the expression levels of IL-1β and TNF-α in both cortex and hippocampus tissues after RNS, implying that IL-33 may exert anti-inflammatory effect partially through blocking NF-κB activation after RNS.

Taken together, the current data demonstrate that IL-33 provides neuroprotection against RNS-induced brain injury through suppressing apoptosis, endoplasmic reticulum stress, and inflammatory pathways, and IL-33 may be a potential therapeutic agent for RNS.

## Data Availability Statement

The original contributions presented in the study are included in the article/supplementary material, further inquiries can be directed to the corresponding authors.

## Ethics Statement

The animal study was reviewed and approved by the NIH Guide for the Care and Use of Laboratory Animals and approved by the Institutional Animal Care and Use Committee at Wenzhou Medical University.

## Author Contributions

YG, CL, and LT established the RNS model, carried out molecular biology experiments, analyzed the data, and wrote the manuscript. JHu, YY, and CX conducted animal behavior experiments. HF and YY performed ELISA experiments. YF, LY, GY, and JHa helped collect tissue samples. All authors have contributed significantly to the design of this experiment, participated in the drafting and rigorous review of this manuscript, and endorsed its final version.

## Conflict of Interest

The authors declare that the research was conducted in the absence of any commercial or financial relationships that could be construed as a potential conflict of interest.

## Abbreviations

BWG: bodyweight gain
CC-3: cleaved-caspase-3
CHOP: CATT/EBP homologous protein
Drp1: dynamin-related protein
ELISA: enzyme-linked immune-sorbent assay
ERS: endoplasmic reticulum stress
GRP-78: glucose-regulated protein 78
HSP70: heat shock protein 70
IL: interleukin
MWM: morris water maze
RNS: recurrent neonatal seizure
ST2: growth stimulation expressed gene 2
SD: Sprague Dawley
TNF- α: tumor necrosis factor- α
WB: western blot.

## References

[B1] Allan D. Fairlie-Clarke K. J. Elliott C. Schuh C. Barnett S. C. Lassmann H. (2016). Role of IL-33 and ST2 signalling pathway in multiple sclerosis: expression by oligodendrocytes and inhibition of myelination in central nervous system. *Acta Neuropathol. Commun.* 4:75. 10.1186/s40478-016-0344-1 27455844 PMC4960877

[B2] Benz A. P. Niquet J. Wasterlain C. G. Rami A. (2014). Status epilepticus in the immature rodent brain alters the dynamics of autophagy. *Curr. Neurovasc. Res.* 11 125–135. 10.2174/1567202611666140305215009 24597603

[B3] Berg A. T. Jallon P. Preux P. M. (2013). The epidemiology of seizure disorders in infancy and childhood: definitions and classifications. *Handb. Clin. Neurol.* 111 391–398. 10.1016/B978-0-444-52891-9.00043-49 23622188

[B4] Brooks C. Cho S. G. Wang C. Y. Yang T. Dong Z. (2011). Fragmented mitochondria are sensitized to Bax insertion and activation during apoptosis. *Am. J. Physiol. Cell Physiol.* 300 C447–C455. 10.1152/ajpcell.00402.2010 21160028 PMC3063970

[B5] Cassidy-Stone A. Chipuk J. E. Ingerman E. Song C. Yoo C. Kuwana T. (2008). Chemical inhibition of the mitochondrial division dynamin reveals its role in Bax/Bak-dependent mitochondrial outer membrane permeabilization. *Dev. Cell* 14 193–204. 10.1016/j.devcel.2007.11.019 18267088 PMC2267902

[B6] Corvino V. Marchese E. Giannetti S. Lattanzi W. Bonvissuto D. Biamonte F. (2012). The neuroprotective and neurogenic effects of neuropeptide Y administration in an animal model of hippocampal neurodegeneration and temporal lobe epilepsy induced by trimethyltin. *J. Neurochem.* 122 415–426. 10.1111/j.1471-4159.2012.07770.x 22537092

[B7] Enatsu R. Mikuni N. (2016). Invasive evaluations for epilepsy surgery: a review of the literature. *Neurol. Med. Chir.* 56 221–227. 10.2176/nmc.ra.2015-2319 PMC4870176 26948700

[B8] Engel T. Caballero-Caballero A. Schindler C. K. Plesnila N. Strasser A. Prehn J. H. (2010). BH3-only protein Bid is dispensable for seizure-induced neuronal death and the associated nuclear accumulation of apoptosis-inducing factor. *J. Neurochem.* 115 92–101. 10.1111/j.1471-4159.2010.06909.xJNC6909 20646170

[B9] Figueiredo C. P. Barros-Aragao F. G. Q. Neris R. L. S. Frost P. S. Soares C. Souza I. N. O. (2019). Zika virus replicates in adult human brain tissue and impairs synapses and memory in mice. *Nat. Commun.* 10:3890. 10.1038/s41467-019-11866-7 31488835 PMC6728367

[B10] Fisher R. S. (2015). Redefining epilepsy. *Curr. Opin. Neurol.* 28 130–135. 10.1097/WCO.0000000000000174 25734953

[B11] Frank S. Gaume B. Bergmann-Leitner E. S. Leitner W. W. Robert E. G. Catez F. (2001). The role of dynamin-related protein 1, a mediator of mitochondrial fission, in apoptosis. *Dev. Cell* 1 515–525. 10.1016/s1534-5807(01)00055-7 11703942

[B12] Fujikawa D. G. Shinmei S. S. Cai B. (2000). Kainic acid-induced seizures produce necrotic, not apoptotic, neurons with internucleosomal DNA cleavage: implications for programmed cell death mechanisms. *Neuroscience* 98 41–53. 10.1016/s0306-4522(00)00085-3 10858610

[B13] Gao Y. Luo C. L. Li L. L. Ye G. H. Gao C. Wang H. C. (2017a). IL-33 provides neuroprotection through suppressing apoptotic, autophagic and NF-kappaB-mediated inflammatory pathways in a rat model of recurrent neonatal seizure. *Front. Mol. Neurosci.* 10:423. 10.3389/fnmol.2017.00423 29311813 PMC5742123

[B14] Gao Y. Ma L. Luo C. L. Wang T. Zhang M. Y. Shen X. (2017b). IL-33 exerts neuroprotective effect in mice intracerebral hemorrhage model through suppressing inflammation/apoptotic/autophagic pathway. *Mol. Neurobiol.* 54 3879–3892. 10.1007/s12035-016-9947-6 27405469

[B15] Gao Y. Zhang M. Y. Wang T. Fan Y. Y. Yu L. S. Ye G. H. (2018). IL-33/ST2L signaling provides neuroprotection through inhibiting autophagy, endoplasmic reticulum stress, and apoptosis in a mouse model of traumatic brain Injury. *Front. Cell Neurosci.* 12:95. 10.3389/fncel.2018.00095 29922130 PMC5996884

[B16] Gorman A. M. Healy S. J. Jager R. Samali A. (2012). Stress management at the ER: regulators of ER stress-induced apoptosis. *Pharmacol. Ther.* 134 306–316. 10.1016/j.pharmthera.2012.02.003 22387231

[B17] Grilli M. Pizzi M. Memo M. Spano P. (1996). Neuroprotection by aspirin and sodium salicylate through blockade of NF-kappaB activation. *Science* 274 1383–1385. 10.1126/science.274.5291.1383 8910280

[B18] He M. Jiang X. Zou Z. Qin X. Zhang S. Guo Y. (2020). Exposure to carbon black nanoparticles increases seizure susceptibility in male mice. *Nanotoxicology* 14 595–611. 10.1080/17435390.2020.1728412 32091294

[B19] Henshall D. C. Simon R. P. (2005). Epilepsy and apoptosis pathways. *J. Cereb. Blood Flow Metab.* 25 1557–1572. 10.1038/sj.jcbfm.9600149 15889042

[B20] Huang L. T. Li H. Sun Q. Liu M. Li W. D. Li S. (2015). IL-33 expression in the cerebral cortex following experimental subarachnoid hemorrhage in rats. *Cell Mol. Neurobiol.* 35 493–501. 10.1007/s10571-014-0143-149 25417195 PMC11486190

[B21] Iwahana H. Yanagisawa K. Ito-Kosaka A. Kuroiwa K. Tago K. Komatsu N. (1999). Different promoter usage and multiple transcription initiation sites of the interleukin-1 receptor-related human ST2 gene in UT-7 and TM12 cells. *Eur. J. Biochem.* 264 397–406. 10.1046/j.1432-1327.1999.00615.x 10491084

[B22] Jafarzadeh A. Mahdavi R. Jamali M. Hajghani H. Nemati M. Ebrahimi H. A. (2016). Increased concentrations of interleukin-33 in the serum and cerebrospinal fluid of patients with multiple sclerosis. *Oman. Med. J.* 31 40–45. 10.5001/omj.2016.08 26813806 PMC4720939

[B23] Jiang H. R. Milovanovic M. Allan D. Niedbala W. Besnard A. G. Fukada S. Y. (2012). IL-33 attenuates EAE by suppressing IL-17 and IFN-gamma production and inducing alternatively activated macrophages. *Eur. J. Immunol.* 42 1804–1814. 10.1002/eji.201141947 22585447

[B24] Jones J. E. Siddarth P. Gurbani S. Shields W. D. Caplan R. (2010). Cognition, academic achievement, language, and psychopathology in pediatric chronic epilepsy: short-term outcomes. *Epilepsy Behav.* 18 211–217. 10.1016/j.yebeh.2010.03.015 20471326 PMC2902590

[B25] Kakkar R. Lee R. T. (2008). The IL-33/ST2 pathway: therapeutic target and novel biomarker. *Nat. Rev. Drug Discov.* 7 827–840. 10.1038/nrd2660 18827826 PMC4277436

[B26] Korotkov A. Broekaart D. W. M. Banchaewa L. Pustjens B. van Scheppingen J. Anink J. J. (2020). microRNA-132 is overexpressed in glia in temporal lobe epilepsy and reduces the expression of pro-epileptogenic factors in human cultured astrocytes. *Glia* 68 60–75. 10.1002/glia.23700 31408236 PMC6899748

[B27] Liu B. Tai Y. Achanta S. Kaelberer M. M. Caceres A. I. Shao X. (2016). IL-33/ST2 signaling excites sensory neurons and mediates itch response in a mouse model of poison ivy contact allergy. *Proc. Natl. Acad. Sci. U.S.A.* 113 E7572–E7579. 10.1073/pnas.1606608113 27821781 PMC5127381

[B28] Liu M. Q. Chen Z. Chen L. X. (2016). Endoplasmic reticulum stress: a novel mechanism and therapeutic target for cardiovascular diseases. *Acta Pharmacol. Sin.* 37 425–443. 10.1038/aps.2015.145 26838072 PMC4820795

[B29] Ljubkovic M. Gressette M. Bulat C. Cavar M. Bakovic D. Fabijanic D. (2019). Disturbed fatty acid oxidation, endoplasmic reticulum stress, and apoptosis in left ventricle of patients with type 2 diabetes. *Diabetes Metab. Res. Rev.* 68 1924–1933. 10.2337/db19-0423 31391173

[B30] Logsdon A. F. Lucke-Wold B. P. Nguyen L. Matsumoto R. R. Turner R. C. Rosen C. L. (2016). Salubrinal reduces oxidative stress, neuroinflammation and impulsive-like behavior in a rodent model of traumatic brain injury. *Brain Res.* 1643 140–151. 10.1016/j.brainres.2016.04.063 27131989 PMC5578618

[B31] Lopez-Meraz M. L. Niquet J. Wasterlain C. G. (2010). Distinct caspase pathways mediate necrosis and apoptosis in subpopulations of hippocampal neurons after status epilepticus. *Epilepsia* 51(Suppl. 3), 56–60. 10.1111/j.1528-1167.2010.02611.x 20618402 PMC2909011

[B32] Molofsky A. B. Savage A. K. Locksley R. M. (2015). Interleukin-33 in tissue homeostasis. injury, and inflammation. *Immunity* 42 1005–1019. 10.1016/j.immuni.2015.06.006 26084021 PMC4471869

[B33] Mychasiuk R. Hehar H. van Waes L. Esser M. J. (2015). Diet, age, and prior injury status differentially alter behavioral outcomes following concussion in rats. *Neurobiol. Dis.* 73 1–11. 10.1016/j.nbd.2014.09.003 25270295

[B34] Noe F. M. Polascheck N. Frigerio F. Bankstahl M. Ravizza T. Marchini S. (2013). Pharmacological blockade of IL-1beta/IL-1 receptor type 1 axis during epileptogenesis provides neuroprotection in two rat models of temporal lobe epilepsy. *Neurobiol. Dis.* 59 183–193. 10.1016/j.nbd.2013.07.015 23938763

[B35] Odegaard J. I. Lee M. W. Sogawa Y. Bertholet A. M. Locksley R. M. Weinberg D. E. (2016). Perinatal Licensing of Thermogenesis by IL-33 and ST2. *Cell* 166 841–854. 10.1016/j.cell.2016.06.040 27453471 PMC4985267

[B36] Palmer G. Talabot-Ayer D. Lamacchia C. Toy D. Seemayer C. A. Viatte S. (2009). Inhibition of interleukin-33 signaling attenuates the severity of experimental arthritis. *Arthritis Rheum.* 60 738–749. 10.1002/art.24305 19248109

[B37] Paschen W. Frandsen A. (2001). Endoplasmic reticulum dysfunction–a common denominator for cell injury in acute and degenerative diseases of the brain? *J. Neurochem.* 79 719–725. 10.1046/j.1471-4159.2001.00623.x 11723164

[B38] Paschen W. Mengesdorf T. (2005). Endoplasmic reticulum stress response and neurodegeneration. *Cell Calcium.* 38 409–415. 10.1016/j.ceca.2005.06.019 16087231

[B39] Qiu X. Cao L. Yang X. Zhao X. Liu X. Han Y. (2013). Role of mitochondrial fission in neuronal injury in pilocarpine-induced epileptic rats. *Neuroscience* 245 157–165. 10.1016/j.neuroscience.2013.04.019 23597828

[B40] Rubovitch V. Barak S. Rachmany L. Goldstein R. B. Zilberstein Y. Pick C. G. (2015). The neuroprotective effect of salubrinal in a mouse model of traumatic brain injury. *Neuromolecular Med.* 17 58–70. 10.1007/s12017-015-8340-8343 25582550

[B41] Russo M. V. McGavern D. B. (2016). Inflammatory neuroprotection following traumatic brain injury. *Science* 353 783–785. 10.1126/science.aaf6260 27540166 PMC5260471

[B42] Sangiuliano B. Perez N. M. Moreira D. F. Belizario J. E. (2014). Cell death-associated molecular-pattern molecules: inflammatory signaling and control. *Mediators Inflamm.* 2014:821043. 10.1155/2014/821043 25140116 PMC4130149

[B43] Scott J. C. McManus D. P. (1999). Identification of novel 70-kDa heat shock protein-encoding cDNAs from *Schistosoma japonicum*. *Int. J. Parasitol.* 29 437–444. 10.1016/s0020-7519(98)00227-6 10333327

[B44] Shin K. H. Yang S. H. Lee J. Y. Lim C. W. Lee S. C. Brown J. W. (2015). Alternative splicing of mini-exons in the arabidopsis leaf rust receptor-like kinase LRK10 genes affects subcellular localisation. *Plant Cell Rep.* 34 495–505. 10.1007/s00299-014-1729-x 25510357

[B45] Sitges M. Gomez C. D. Aldana B. I. (2014). Sertraline reduces IL-1beta and TNF-alpha mRNA expression and overcomes their rise induced by seizures in the rat hippocampus. *PLoS One* 9:e111665. 10.1371/journal.pone.0111665 25364907 PMC4218797

[B46] Sola-Riera C. Gupta S. Maleki K. T. Gonzalez-Rodriguez P. Saidi D. Zimmer C. L. (2019). Hantavirus inhibits TRAIL-mediated killing of infected cells by downregulating death receptor 5. *Cell Rep.* 28 2124.e6–2139.e6. 10.1016/j.celrep.2019.07.066 31433987

[B47] Sousa C. (2013). Valproic acid-induced hyperammonemic encephalopathy - a potentially fatal adverse drug reaction. *Springerplus* 2:13. 10.1186/2193-1801-2-13 23451336 PMC3579419

[B48] van der Heide M. J. Roze E. van der Veere C. N. Ter Horst H. J. Brouwer O. F. Bos A. F. (2012). Long-term neurological outcome of term-born children treated with two or more anti-epileptic drugs during the neonatal period. *Early. Hum. Dev.* 88 33–38. 10.1016/j.earlhumdev.2011.06.012 21835564

[B49] Vince J. E. Silke J. (2016). The intersection of cell death and inflammasome activation. *Cell Mol. Life. Sci.* 73 2349–2367. 10.1007/s00018-016-2205-2202 27066895 PMC11108284

[B50] Xiao Z. Peng J. Yang L. Kong H. Yin F. (2015). Interleukin-1beta plays a role in the pathogenesis of mesial temporal lobe epilepsy through the PI3K/Akt/mTOR signaling pathway in hippocampal neurons. *J. Neuroimmunol.* 282 110–117. 10.1016/j.jneuroim.2015.04.003 25903737

[B51] Xie N. Wang C. Wu C. Cheng X. Gao Y. Zhang H. (2016). Mdivi-1 protects epileptic hippocampal neurons from apoptosis via inhibiting oxidative stress and endoplasmic reticulum stress in vitro. *Neurochem. Res.* 41 1335–1342. 10.1007/s11064-016-1835-y 26801176

[B52] Yakovlev A. G. Faden A. I. (2001). Caspase-dependent apoptotic pathways in CNS injury. *Mol. Neurobiol.* 24 131–144. 10.1385/MN:24:1-3 11831549

[B53] Yamamoto A. Murphy N. Schindler C. K. So N. K. Stohr S. Taki W. (2006). Endoplasmic reticulum stress and apoptosis signaling in human temporal lobe epilepsy. *J. Neuropathol. Exp. Neurol.* 65 217–225. 10.1097/01.jnen.0000202886.22082.2a 16651883

[B54] Yang Y. Liu H. Zhang H. Ye Q. Wang J. Yang B. (2017). ST2/IL-33-dependent microglial response limits acute ischemic brain injury. *J. Neurosci.* 37 4692–4704. 10.1523/JNEUROSCI.3233-16.2017 28389473 PMC5426564

[B55] Yu M. H. Choi J. H. Chae I. G. Im H. G. Yang S. A. More K. (2013). Suppression of LPS-induced inflammatory activities by Rosmarinus officinalis L. *Food Chem.* 136 1047–1054. 10.1016/j.foodchem.2012.08.085 23122161

[B56] Yue J. He J. Wei Y. Shen K. Wu K. Yang X. (2020). Decreased expression of Rev-Erbalpha in the epileptic foci of temporal lobe epilepsy and activation of Rev-Erbalpha have anti-inflammatory and neuroprotective effects in the pilocarpine model. *J. Neuroinflammation* 17:43. 10.1186/s12974-020-1718-7 32005256 PMC6993411

[B57] Zarpelon A. C. Rodrigues F. C. Lopes A. H. Souza G. R. Carvalho T. T. Pinto L. G. (2016). Spinal cord oligodendrocyte-derived alarmin IL-33 mediates neuropathic pain. *FASEB J.* 30 54–65. 10.1096/fj.14-267146 26310268

[B58] Zhang J. Ney P. A. (2008). NIX induces mitochondrial autophagy in reticulocytes. *Autophagy* 4 354–356. 10.4161/auto.5552 18623629

[B59] Zhou B. H. Wei S. S. Jia L. S. Zhang Y. Miao C. Y. Wang H. W. (2020). Drp1/Mff signaling pathway is involved in fluoride-induced abnormal fission of hepatocyte mitochondria in mice. *Sci. Total Environ.* 725:138192. 10.1016/j.scitotenv.2020.138192 32278173

[B60] Ziegler D. R. Araujo E. Rotta L. N. Perry M. L. Goncalves C. A. (2002). A ketogenic diet increases protein phosphorylation in brain slices of rats. *J. Nutr.* 132 483–487. 10.1093/jn/132.3.483 11880575

